# Can Archival Tissue Reveal Answers to Modern Research Questions?: Computer-Aided Histological Assessment of Neuroblastoma Tumours Collected over 60 Years

**DOI:** 10.3390/microarrays3010072

**Published:** 2014-02-28

**Authors:** Albert Chetcuti, Nicole Mackie, Siamak Tafavogh, Nicole Graf, Tony Henwood, Amanda Charlton, Daniel Catchpoole

**Affiliations:** 1 Tumour Bank, The Children’s Cancer Research Unit, Kid’s Research Institute, The Children’s Hospital at Westmead, Westmead, NSW 2145, Australia; E-Mail: a.chetcuti@hotmail.com; 2 Histopathology Department, The Children’s Hospital at Westmead, Westmead, NSW 2145, Australia; E-Mails: nmackie7@gmail.com (N.M.); nicole.graf@health.nsw.gov.au (N.G.); tony.henwood@health.nsw.gov.au (T.H.); amanda.charlton@health.nsw.gov.au (A.C.); 3 Faculty of Engineering and Information Technology, The University of Technology Sydney, Sydney, NSW 2007, Australia; E-Mail: siamak.tafavogh@student.uts.edu.au

**Keywords:** tissue microarray, archival tissue, neuroblastoma, immunohistochemistry, image analysis

## Abstract

Despite neuroblastoma being the most common extracranial solid cancer in childhood, it is still a rare disease. Consequently, the unavailability of tissue for research limits the statistical power of studies. Pathology archives are possible sources of rare tissue, which, if proven to remain consistent over time, could prove useful to research of rare disease types. We applied immunohistochemistry to investigate whether long term storage caused any changes to antigens used diagnostically for neuroblastoma. We constructed and quantitatively assessed a tissue microarray containing neuroblastoma archival material dating between 1950 and 2007. A total of 119 neuroblastoma tissue cores were included spanning 6 decades. Fourteen antibodies were screened across the tissue microarray (TMA). These included seven positive neuroblastoma diagnosis markers (NB84, Chromogranin A, NSE, Ki-67, INI1, Neurofilament Protein, Synaptophysin), two anticipated to be negative (S100A, CD99), and five research antibodies (IL-7, IL-7R, JAK1, JAK3, STAT5). The staining of these antibodies was evaluated using Aperio ImageScope software along with novel pattern recognition and quantification algorithms. This analysis demonstrated that marker signal intensity did not decrease over time and that storage for 60 years had little effect on antigenicity. The construction and assessment of this neuroblastoma TMA has demonstrated the feasibility of using archival samples for research.

## 1. Introduction

Neuroblastoma is the most common extracranial solid tumour in childhood and the most common cancer found in children under the age of five [1,2]. It is considered that neuroblastoma arises when the differentiation of these migratory cells of the neural crest are blocked [3,4,5,6]. The differences in outcome for patients with neuroblastoma are striking. Infants younger than 1 year have a good prognosis, even in the presence of metastatic disease, whereas older patients with advanced stage neuroblastoma fare poorly, despite aggressive multimodality therapy [7,8]. Fewer than half of these patients are cured, even with the use of high-dose therapy followed by autologous bone marrow or stem cell rescue [8]. The molecular basis underlying the variability in tumour growth, clinical behaviour, and responsiveness to therapy remains largely unknown [9]. However, the review by Riley and co-authors [10] identify a range of informative molecular and biological markers that are now routinely used to direct the clinical management and treatment of neuroblastoma patients.

When compared to adult cancers, neuroblastoma is a very rare disease with current figures in Australia indicating that approximately 35–40 new cases are diagnosed each year [11]. With these relatively low numbers, obtaining sufficient samples to conduct research is challenging in countries like Australia with a relatively low population. Translational research requires suitable collections of tissue samples to be made available for these investigations, yet whilst samples collected from multiple institutes to accumulate sufficient numbers may be feasible, quality assurance requirements many not be guaranteed across cohorts collected from institutions that have diverse biobanking practices.

Archived formalin-fixed paraffin-embedded (FFPE) tissues stored in hospital histopathology departments represent valuable collections of biospecimens that allow modern research questions to be investigated in rare tumours. It is queried, however, whether biospecimens prepared and stored over many decades allow a range of tests for protein and DNA elements to be performed to a similar standard as those prepared using current protocols [12,13,14,15]. As the consistency of laboratory results obtained from samples stored over lengthy periods of time is one of the major concerns of biobanks, we compared the marker staining results from FFPE biopsies of neuroblastoma tumours stored between 1950 and 2007 within a single paediatric hospital. To enable the direct comparison of such a diverse collection of samples, a tissue microarray (TMA) containing all materials tested was constructed [16]. Array sections were immunohistochemically (IHC) evaluated for current positive and negative diagnostic markers as well as with novel research-specific antibodies. It was reasoned that protein antigenicity throughout the tissue section could be lost over time. In this context, our principal measure for staining quality was signal intensity, which, by using tissue microarrays, can be systematically and objectively compared across multiple samples treated under exactly the same experimental conditions. Computer-aided digital analysis has been applied to quantitatively determine signal intensity for the antibodies used in each tissue sample on the array [17,18,19,20].

We have demonstrated that despite long term storage of these neuroblastoma specimens, the tissues performed to an equivalent level, and thus open the potential for a large amount of archival material to be used for research purposes into rare disease.

## 2. Experimental Section

### 2.1. Neuroblastoma Cases and TMA Construction

**Figure 1:**
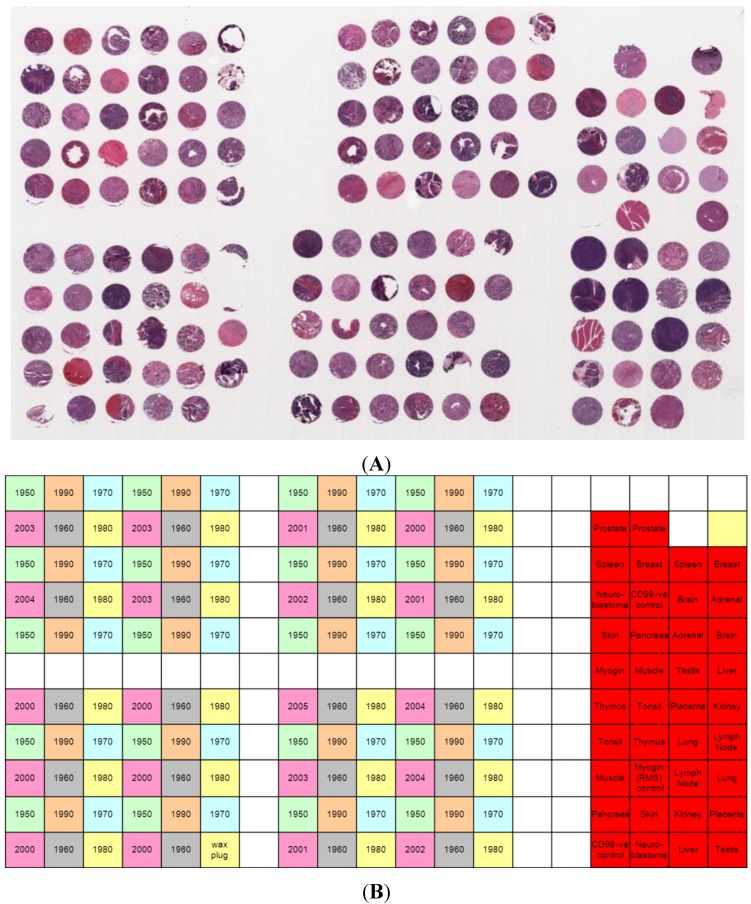
(**A**) Haematoxylin and eosin stained section of the TMA, containing 119 neuroblastoma tissue cores from 49 individuals and 38 control tissues. (**B**) Map of the TMA layout. A variety of normal and control tissues were included.

An extensive search of The Children’s Hospital at Westmead Histopathology department records was conducted, and established a total of 422 neuroblastoma cases treated between 1950 and 2007 for which archival paraffin embedded material was stored. Individual neuroblastoma cases with sufficient viable tumour tissue were assessed by clinical pathologists for inclusion in the tissue microarray. Tissue processing conditions, including fixative and fixation time, were sought from the hospital records, but these were incomplete. Forty nine chosen samples were de-identified to maintain patient privacy and meet local legal and ethical requirements. Neuroblastoma cases were separated in decades (1950–1959, 1960–1969, 1970–1979, 1980–1981, 1990–1999, and 2000–2007) and cases were selected for inclusion in the TMA if sufficient material to include 2–4 cores/case was possible. A total of 20 cores per decade were included in the TMA layout; 120 samples in total (Figure 1). Neuroblastoma tissue cores from the same decade were distributed evenly across the TMA in a 4 × (6 × 5 array) block pattern (Figure 1B). 

Normal control and control tumour tissues were also included in the TMA. These included the following tissue types: liver, testis, pancreas, skin, kidney, placenta, lung, lymph node, muscle, tonsil, thymus, brain, adrenal, breast, spleen, prostate, rhabdomyosarcoma, and Ewing’s sarcoma. Each control tissue was included in duplicate. The TMA was constructed using a Manual Tissue Arrayer MTA-1 (Beecher Instruments, WI, USA) by following the standard instructions [21]. All cores included in the TMA were 1.0 mm in diameter. A donor paraffin block was prepared using paraplast wax (Leica Microsystems, Sydney, Australia) and cored prior to the addition of each recipient tissue core. Following the completion of the TMA, it was annealed using the following procedure: The TMA block was annealed via five cycles of heating at 60 °C for 10 min and cooling at room temperature for 20 min. Following each heating and cooling cycle, the TMA block surface was lightly pressed by hand with a clean glass slide. Serial sections from the TMA block were cut at a thickness of 4 µm using a standard microtome, mounted onto positively charged Superfrost glass microscope slides (Menzel-Glaser, Germany) and dried in at 65 °C. Every tenth section was stained with haematoxylin and eosin using a Vision Biosystems Autostainer (Leica Microsystems) to assess the quality of the cores throughout the TMA block.

### 2.2. Immunohistochemistry

All immunohistochemical staining was performed using the Bond Max Vision BioSystems (Leica Microsystems) automated immunohistochemistry stainer. The following primary antibodies and dilutions were used: Neuroblastoma Marker (clone NB84a, Leica Microsystems) at 1/1000 dilution; Chromogranin A (clone LK2H10, Leica Microsystems) at 1/200 dilution; Neuron Specific Enolase (NSE) (clone 22C9, Leica Microsystems) at 1/100 dilution; Ki-67 (clone MIB-1, Dako, Melbourne, Australia) at 1/200 dilution; INI1 (clone BAF47, BD Biosciences, Sydney, Australia) at 1/500 dilution; Neurofilament Protein (clone 2F11, Dako) at 1/1600 dilution; S100 (clone Z0311, Dako) at 1/1000 dilution; CD99 (clone 12E7, Leica Microsystems) at 1/1200 dilution; Synaptophysin (clone Z66, Invitrogen, Melbourne, Australia) at 1/150 dilution; Interleukin-7 (clone H-151, Santa Cruz Biotechnology, Santa Cruz, CA, USA) at 1/50 dilution; Interleukin-7 receptor (clone H-215, Santa Cruz Biotechnology) at 1/50 dilution, Janus kinase 1 (clone H-106, Santa Cruz Biotechnology) at 1/50 dilution, Janus kinase 3 (clone B-12, Santa Cruz Biotechnology) at 1/50 dilution; and Signal transducer and activator of transcription 5 (clone N-20, Santa Cruz Biotechnology) at 1/50 dilution.

The following antigen retrieval methods were used: For Chromagranin A, Ki-67, INI1, Neurofilament Protein, and CD99 antibodies, heat induced epitope retrieval was performed using the Bond Epitope Retrieval Solution 1 (AR9961, Leica Microsystems). For the Synaptophysin, IL-7, IL-7R, JAK1, JAK3, and STAT5 antibodies heat induced epitope retrieval was performed using the Bond Epitope Retrieval Solution 2 (AR9640, Leica Microsystems). For the NB84 antibody, a pronase enzyme retrieval method was used. No antigen retrieval was used for the NSE or S100 primary antibodies. Primary antibody binding was detected using the Bond Polymer Refine Detection Kit (Leica Microsystems). Slides were counterstained with hematoxylin, cover slipped and permanently mounted using Histomount (National Diagnostics, Adelaide, Australia).

### 2.3. Evaluation of TMA Immunohistochemistry

#### 2.3.1. Digital Image Acquisition

Stained TMA slides were scanned on an Aperio ScanScope CS virtual microscope (Aperio, CA, USA). Each stained tissue microarray slide was scanned at 400× absolute magnification to yield high resolution (0.25 μm^2^/pixel) images. 

#### 2.3.2. Computer-Aided Image Analysis: Cell Counting

To determine whether each tissue disc contained equivalent numbers of tumour cells, we applied two imaging algorithms developed by and previously described us [19,20] for images of haematoxylin and eosin stained tissue array slides. Briefly, the first algorithm enhances the fluctuating intensity quality of the images by reducing the wide range of colour variation within the images. It partitions the image into several mosaics and normalises the colour of all the pixels within each mosaic, thereby reducing the sensitivity of the downstream analysis to noisy images and improving their performance when identifying and extracting the regions of interests. The second algorithm exploits the differences between the colours, morphological features, and geometrical properties to identify and extract histological elements, such as cellular regions, areas of stroma and neuropil, red blood cells, and background spaces. Cellular regions and areas of stroma/neuropil are presented as percentages of the total area covered by tissue spot.

#### 2.3.3. Computer-Aided Image Analysis: Pixel Counting for Signal Intensity

**Figure 2:**
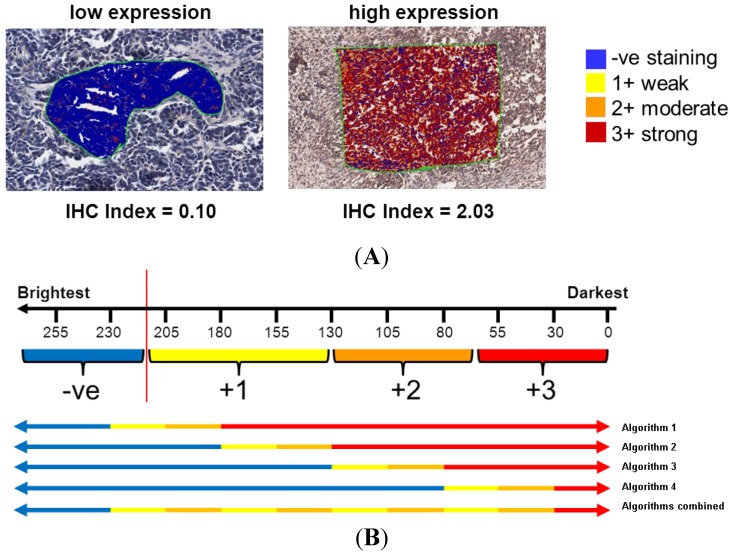
(**A**) Examples showing mark up areas for positive pixel counting with indicative IHC index. Colours represent the bin (negative–strong) with which a pixel is placed, depending on the intensity of light transmitted through the slide at that point. (**B**) To determine the distribution of staining intensity across the image, the algorithm was used four times, shifting the binning constraints of the positive pixel counting each time to segregate the pixel distribution into 10 subdivisions. A high value (>230) represents no antibody staining, whilst a low value (<30) represents maximal antibody staining.

Optimised positive pixel counting algorithms (v9.1, Aperio, Vista, CA, USA) were used for digital analysis, and measured using ImageScope v10.2.2.2352 software (Aperio). An index was calculated to determine the overall signal intensity for all individual tissue spots for each IHC antibody stain based on previously reported approaches [22,23]. The IHC index is based on the addition of weighted proportions as follows:

IHC Index = (n_1_/T) × 1 + (n_2_/T) × 2 + (n_3_/T) × 3 (1)

where T is the total number of pixels per spot, n is the number of stained pixels per spot, and the subscripts 1, 2, 3 represent weak, moderate, and strong staining, respectively (Figure 2A). An IHC index of 0.0 indicates no signal and no protein expression, whilst an index of 3.0 indicates that all pixels are strongly positive and expression is high throughout the sample. This approach was modified to determine the distribution of antibody staining signal intensity across each neuroblastoma tissue core. The positive pixel counting algorithm was adjusted so that the ‘weak’ and ‘moderate’ intensity bins would capture only one tenth of a specific signal range. The algorithm was run iteratively with these bin regions shifted across the intensity spectrum until the staining intensity was partitioned into 10 equal subdivisions from negative staining (>230) to maximal positive staining (<30) (Figure 2B). The signal intensity for each subdivision was averaged across each decadal storage period to determine changes to the distribution of antigenicity of selected proteins over time.

### 2.4. Statistical Analysis

For immunohistochemistry results, Pearson’s correlation test was used to compare TMA core staining across the collection time period. A *p*-value of <0.05 was considered statistically significant.

## 3. Results and Discussion

### 3.1. Neuroblastoma TMA Core Morphology

**Figure 3:**
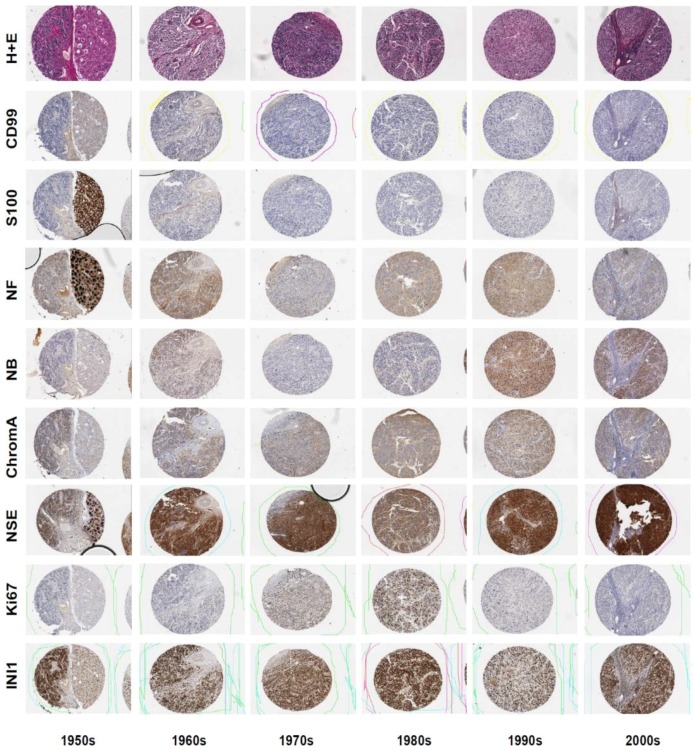
Selection of six neuroblastoma tumours, collected between 1950–2007. Staining with haematoxylin and eosin, and IHC using a range of antibodies demonstrated maintenance of general staining attributes over storage time. A sample from 1950 showed unique histology with half the sample representing differentiated ganglioneuroma morphology (**right**) and half undifferentiated neuroblastoma (**left**).

In this study, it was important to demonstrate that all tissue cores on the array represented the equivalent proportion of cellular and stromal structures, which is commonly typical for neuroblastoma tumours. Hematoxylin and eosin stained TMA sections were visually assessed for general tissue morphology and histological quality (NG, AC, TH). All cores from each time period demonstrated intact cellular architecture and clarity of staining such that all histological structures of the neuroblastoma tumours could be determined (Figure 3). Despite the absence of full records of tissue processing conditions, no gross fixation or processing artefacts were noted.

**Figure 4:**
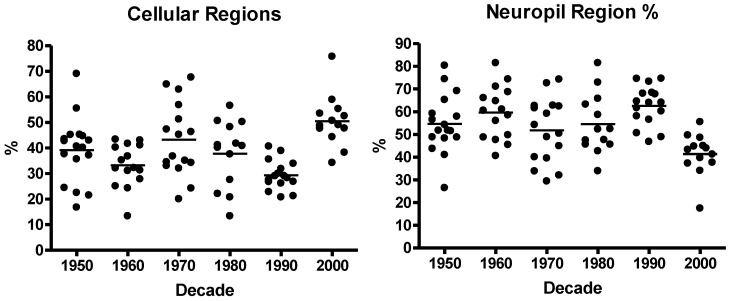
Proportion of cellular (**left**) and stromal/neuropil (**right**) structures within each sample of neuroblastoma tissue collected in each decade (1950–2000).

Quantitative analysis of each core using our specialist pattern recognition algorithms demonstrate that the average percentage of each core represented by either cellular or stroma/neuropil is 38.6 ± 12.7% and 54.4 ± 13.3% respectively. Samples across each decade demonstrated equivalent distribution of cellular proportions, although it is clear that samples selected from the 2000’s were generally more cellular whilst those from the 1990s had more stromal elements represented than other decades (Figure 4). We consider this bias to be a random anomaly within the selection of samples included in the TMA and does not reflect a systematic change in sample processing or surgical practice.

### 3.2. Immunohistochemistry

**Figure 5:**
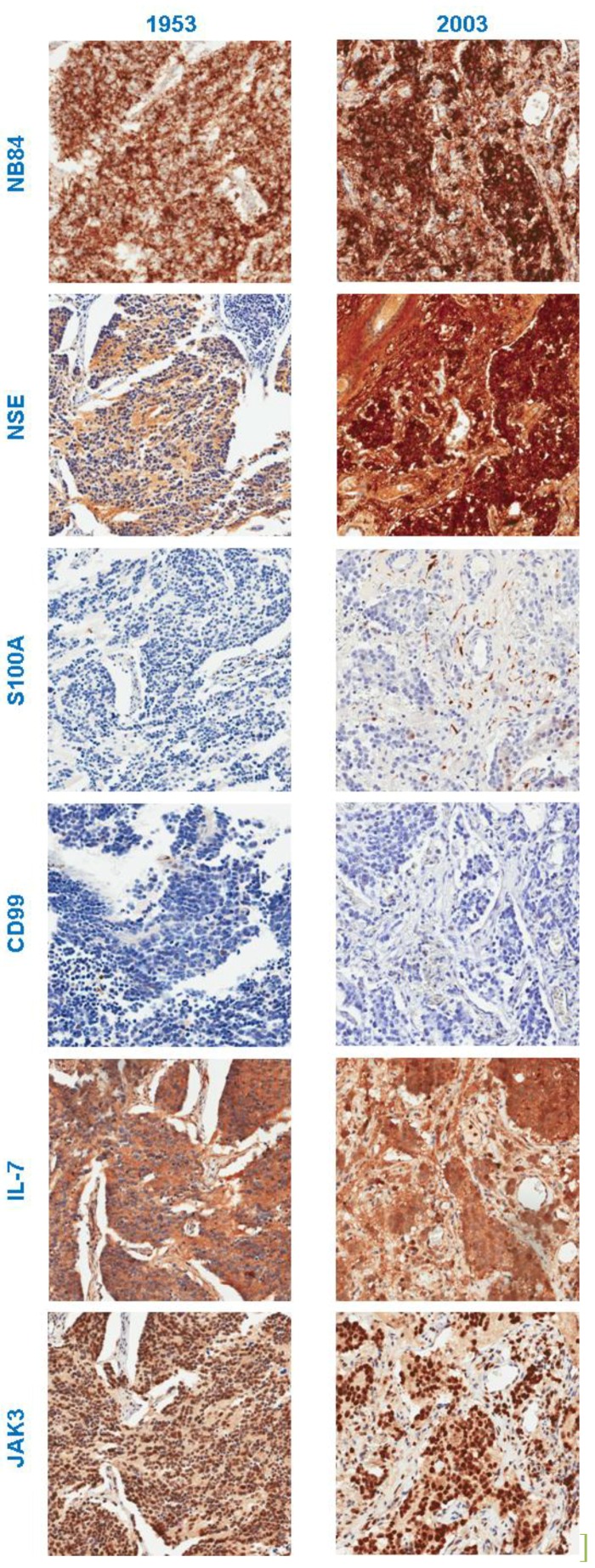
Immunohistochemical staining with antibodies against NB84, NSE, S100A, CD99, IL-7, and JAK3 in neuroblastoma tissue collected in 1953 or 2003.

Having demonstrated the histological equivalency of the tumour samples selected for inclusion on the tissue microarray, the specificity of antibody staining was considered. We compared the staining observed in the control tissue cores as well as the neuroblastoma tissue for each section. Cell specific staining was observed for all antibodies used (Figure 3). As shown in Figure 5, strong staining for the positive diagnosis marker NB84 was seen in old (1953) and modern (2003) neuroblastoma tissue. Conversely, no staining was observed for the negative marker CD99 in old or modern sections (Figure 5). Visually, samples collected in the 1950s demonstrated the least intense positive staining and higher background staining, whilst those from between 1960–1990 performed comparably well with moderate levels of positive staining and weak negative background staining. Generally, samples collected from 2000 onwards demonstrated the most distinct staining pattern with strong positive signals as well as no staining for negative markers. Despite these observed distinctions, all samples performed adequately using modern immunohistochemical methodologies, yielding measurable results.

### 3.3. Computer-Aided Image Analysis

**Figure 6:**
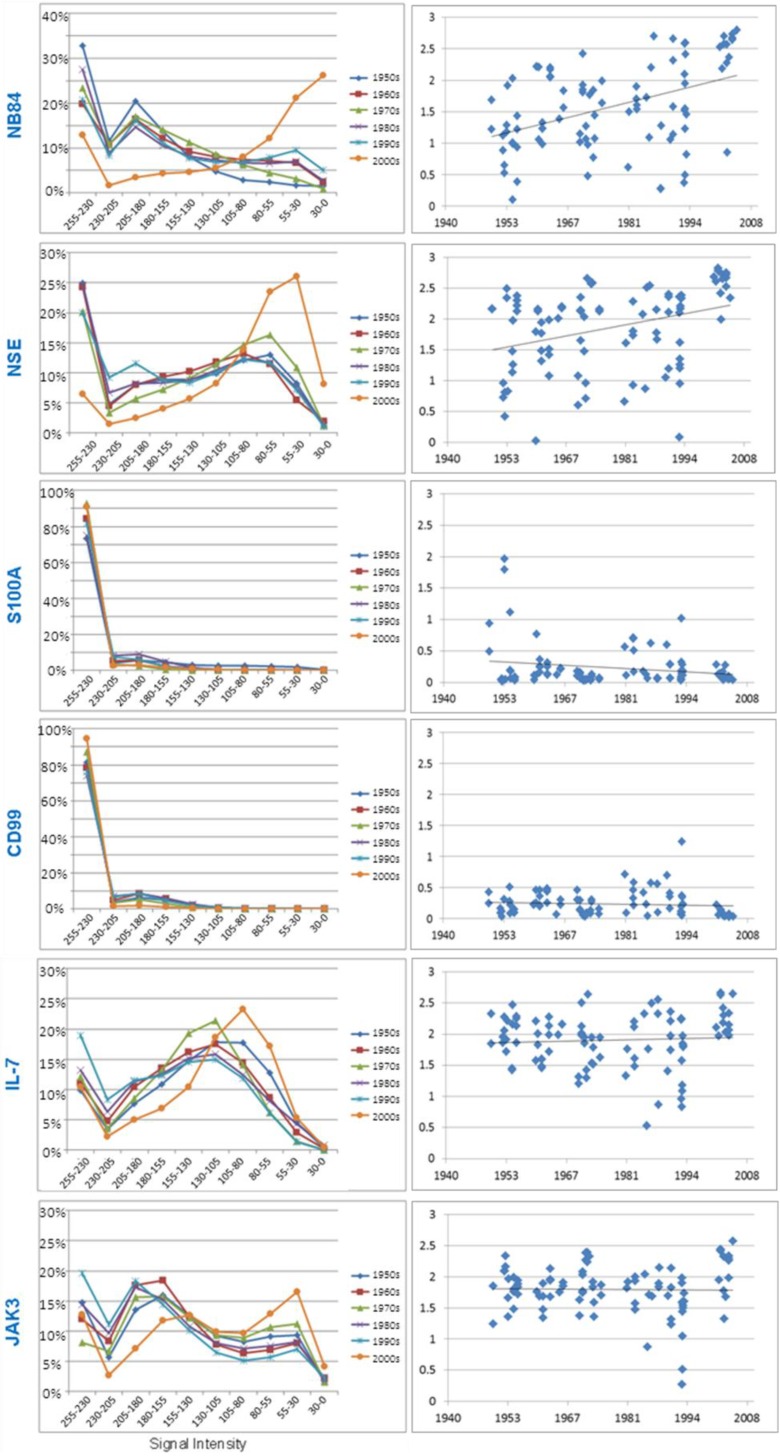
Pearson’s correlation for each neuroblastoma tissue core *versus* collection date. **Left column**: Plot of the average positive pixel signal intensity bins per algorithm subdivision for each decade. **Right Column**: Scatter plots of the IHC index for each tissue core compared to collection date.

To identify trends in antigenicity for each protein *versus* tissue age, the total signal intensity (IHC index) of each TMA tissue core was plotted against collection date (Figure 6), with regression analysis used to explore any changes is antigenicity over time (Table 1). Furthermore, the average distribution of each antibody’s signal across tissue samples collected from each decade is shown in Figure 6. In general, the majority of proteins examined did not demonstrate changes in antigenicity over the 60 year collection period that could be related to a storage related decline in tissue quality. The results of the image analysis for each individual antibody stain are described below.

#### 3.3.1. Neuroblastoma Marker NB84

**Figure 7:**
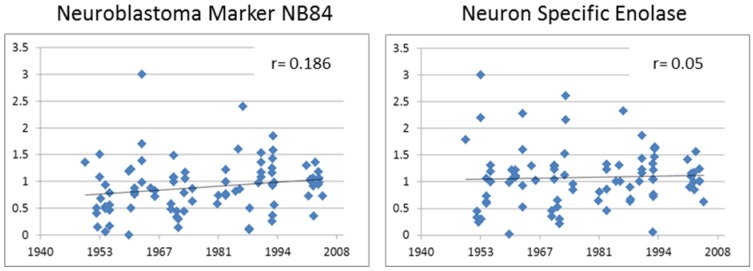
IHC index for NB84 and NSE corrected for cellular content.

Diagnostically, this antibody indicates the presence of neuroblastoma cells. The anti-neuroblastoma marker antibody NB84 stains an uncharacterised 57 kD molecular protein found in most normal human tissues, including epithelial and endothelial cells. This antibody staining was predominately cytoplasmic and medium to strong in all neuroblastoma cores [24]. In control tissue, strong NB84 staining was found in prostate epithelium, liver, kidney, placenta, lung, breast, spleen, and adrenal tissues. Negative NB84 staining was found in muscle, pancreas, skin, rhabdomyosarcoma, thymus, Ewing’s sarcoma, brain, lymph node, and tonsil (data not shown). A statistically significant trend (*p* < 0.001) in the IHC index over time suggested that total antigenicity for NB84 decreased with extended storage (Figure 6(right); Table 1). It must be noted, however, that the newer samples represented on the array were previously shown to be more cellular (Figure 4) and this result may be biased accordingly. Hence, to compensate for this potential bias, we worked with the assumption that all the staining signal for NB84 would be found in the cellular regions and corrected the IHC index for the percentage of the image represented by cells. This resulted in the fact that the trend for a decreasing signal with storage time disappeared (r = 0.186; Figure 7).

The distribution of signal over each core was notably more pronounced towards the darker intensity for the most recent samples (2000’s) compared to all others (Figure 6(left)). This confirmed the results in Figure 5 that the most recent NB84 staining had more contrast, with pixels showing strong to maximal intensity indicative of specific tissue elements being heavily stained. Samples greater than 10 years old, however, demonstrated a more uniform distribution of staining intensity, albeit fainter and more diffuse.

**Table 1:** Pearson’s correlation for antibody antigenicity compared to collection date.

	Marker	Linear Regression (R)	*p* value
Diagnostic	NB84	0.432	<0.001
	Chromogranin A	−0.138	0.144
	NSE	0.209	0.026
	Ki-67	0.177	0.066
	INI7	0.072	0.452
	Neurofilament Protein	−0.050	0.607
	S100	−0.258	0.006
	CD99	−0.200	0.039
	Synaptophysin	−0.109	0.265
Research	IL-7	−0.033	0.724
	IL-7R	0.037	0.698
	JAK1	0.006	0.951
	JAK3	0.065	0.501
	STAT5	−0.018	0.855

#### 3.3.2. Neuron Specific Enolase

The anti-Neuron Specific Enolase (NSE) antibody stains the 47 kD component of the gamma-gamma enolase isoenzyme, which is involved in glycolytic metabolism found in all neurons. This antibody staining was strong in all neuroblastoma cores and was predominately cytoplasmic in nature [25]. In control tissue cores, strong staining was found in brain, adrenal, kidney, rhabdomyosarcoma, spleen, and skin, but was negative in liver, thymus, tonsil, prostate, pancreas, Ewing’s sarcoma, breast, testis, placenta, lung, kidney, and muscle. Like the NB84 results, the IHC index used to quantitate NSE staining demonstrated a significant decreasing trend over time (r = 0.209, *p* = 0.026) (Figure 6(right); Table 1). Similarly, however, when corrected for cellular content of each sample, this decreasing trend was lost (r = 0.05) (Figure 7). The distribution of NSE intensity across each tissue spot was shifted towards greater intensity and contrast in the most recent samples (Figure 6(left)).

#### 3.3.3. S100A

The anti-S100 antibody stains the S100A protein and is a positive marker for Schwann cells of the peripheral nervous system and highlights Schwannian stroma in differentiated ganglioneuroblastoma (Figure 3; 1950’s) as well as sustentacular cells in rare olfactory neuroblastoma [26,27]. This antibody staining is low in undifferentiated ‘stroma-poor’ neuroblastoma tumours that were selected for inclusion in the tissue microarray. The IHC index confirmed low S100 expression in all samples, especially those collected between 1960 and 2005. Although the majority of neuroblastoma cores showed low level S100 staining, samples collected during the 1950s shown some weak to moderate positive staining (Figure 6(right)).

#### 3.3.4. CD99

The anti-CD99 antibody stains a 32 kDa transmembrane glycoprotein, encoded by the MIC2 gene and is used as a diagnostic marker for Ewing’s sarcoma. This antibody, however, stains negative in neuroblastoma cores. In control tissue cores, strong membrane and medium cytoplasmic staining was found in Ewing’s sarcoma, prostate, and spleen tissues, as anticipated [28]. The IHC index demonstrated all neuroblastoma cores as negative for CD99 staining across all decade (Figure 6(right)).

#### 3.3.5. Research Antibodies: Interleukin-7, Interleukin-7 Receptor, Stat5, JAK1, and JAK3

The interleukin-7 protein is a 20 kDa growth factor cytokine that is known to induce neuronal cell differentiation [29] and is purported to have a functional role in neuroblastoma [30]. Members of the IL7 signalling pathway, IL7-receptor, JAK1, JAK3, and Stat5 were also examined across this array. All antibodies showed measurable cellular staining with the IHC index demonstrating consistency in intensity across all neuroblastoma tissue collected over six decades (Table 1). This is exemplified by IL-7 (r = −0.033, *p* = 0.724) and JAK3 (r = 0.065, *p* = 0.501) (Figure 6(right); Table 1). The intensity distribution across each tissue spot for all antibodies was shifted towards greater intensity and contrast in the most recent samples, although this difference was only modest (Figure 6(left)).

#### 3.3.6. Chromogranin A, Ki-67, INI1, Neurofilament Protein and Synaptophysin.

The following additional diagnostic antibodies were investigated: Anti-chromogranin A antibody stains a 68 kD acidic protein, which is widely expressed in neural tissues and neuroendocrine tumours [31,32]. The anti-Ki-67 antibody stains Ki-67, which is a nuclear protein that is preferentially expressed during all active phases of the cell cycle [31]. The anti-Integrase Interactor 1 (INI1) antibody stains the BAF47 component of the SWI/SNF5 complex. SWI/SNF complexes facilitate gene activation and transcription factor binding by altering repressive chromatin structures in an ATP-dependent manner [33]. The anti-Neurofilament Protein antibody stains a 47 kDa BAF (BRG1-associated factors) protein. This antibody labels neurons (axons) of the central and peripheral nervous system and is useful for the identification of tumours with neuronal differentiation [31]. The anti-Synaptophysin antibody stains a 38 kDa glycosylated polypeptide and is a positive neuroblastoma marker [31]. All antibodies produced measurable and observable staining across all neuroblastoma cores. However, no statistically consistent change in staining signal intensity was observed (Figure 4; Table 1).

## 4. Conclusions

The question of ‘tissue quality’ is an undefined and vexed one. Study designs often require all tissue specimens to be collected in the same fashion to exclude any storage or tissue handling activity that can confound later results. Such requirements are difficult to comply with when investigating rare diseases, and low donor numbers limit the statistical power required for modern research. We have demonstrated, using tissue microarray technology, that despite long term storage within a single hospital’s archive, 60 year old paraffin-embedded neuroblastoma specimens retain their antigenicity for currently used diagnostic markers. Our results concur with the findings of Litlekalsoy *et al.* [13] and Camp *et al.* [14], who performed similar studies on individual cohorts of 144 urinary bladder carcinomas or 38 breast carcinomas dating back from the present to 1932. Both concluded that the successful detection of specific proteins will enhance large retrospective investigations into rare diseases. Our study strengthens such findings by using TMA technology and subjecting every slide to exactly the same immunohistochemistry procedure at the same time. Visual assessment of tissue staining was avoided to limit subjectivity in reporting. Therefore, we used computer-aided analysis as our primary tool for measuring staining signal to objectively demonstrate consistency in staining intensity across all ages of sample obtained from a single institute.

Variability in the signal intensity across the arrays was seen; most notably, staining distribution within samples collected most recently, indicating a greater level of signal contrast. However, this change in signal distribution and image quality did not persist over time, with samples older than 10 years generally yielding more consistent results. Time in storage for these FFPE samples does not appear to have a major effect on sample quality, but rather histological techniques, such as sample handling protocols, tissue fixation, and antigen retrieval conditions [34,35], appear to be stronger influences on how tissue stains. Indeed the generally tight clustering of all signals from our most recent samples (2000’s) indicates that present day tissue handling, immunohistochemistry techniques, and antibody quality have tissue improved to such an extent that more standardized results are obtained. These findings add support to the option for researchers to access archival material when conducting large retrospective studies into rare diseases and promise that meaningful answers to modern day questions can be obtained with a high degree of accuracy.
